# Lived Experiences and Long‐Term Reflections on Early Marriage Among Palestinian Women: A Qualitative Study in the West Bank

**DOI:** 10.1002/puh2.70290

**Published:** 2026-05-29

**Authors:** Nesreen Alqaissi, Mohammad Qtait

**Affiliations:** ^1^ Nursing College Palestine Polytechnic University Hebron Palestine

**Keywords:** child marriage, early marriage, gender norms, lived experiences, Palestinian women, psychosocial impact, qualitative research

## Abstract

**Background:**

Early marriage remains a persistent practice in conflict‐affected settings, including the West Bank of Palestine, where sociocultural norms, economic pressures, and political instability intersect. Although prior research has largely focused on short‐term outcomes, limited qualitative evidence examines how women retrospectively interpret early marriage across the life course.

**Objective:**

This study explores the lived experiences and long‐term reflections of Palestinian women married before the age of 18, with particular attention to agency, psychosocial development, and meaning‐making over time.

**Methods:**

A qualitative design using reflexive thematic analysis was employed. Ten Palestinian women (aged 23–35), married between ages 7 and 17, were purposively recruited from the Hebron Governorate. Semi‐structured interviews were conducted in Arabic, transcribed verbatim, and translated with linguistic verification. Data were analyzed iteratively following Braun and Clarke's approach, with attention to theme recurrence, variation, and negative cases. Interpretation was guided by Bronfenbrenner's ecological systems theory and feminist theory.

**Results:**

Three interrelated themes were identified: (1) constrained agency within familial and sociocultural systems, where marriage decisions were shaped by hierarchical power structures; (2) multidimensional and enduring impacts, including physical strain, emotional burden, and social disconnection; and (3) retrospective reappraisal and disrupted life trajectories, characterized by loss of educational opportunities and evolving critical awareness. Variability across participants highlighted differences related to age at marriage and educational pathways.

**Conclusion:**

Findings demonstrate that early marriage operates as a structurally embedded and gendered process shaping women's life courses. Participants’ narratives reflect both constraint and adaptive meaning‐making, underscoring the need for contextually grounded interventions that address intersecting social, cultural, and structural determinants.

## 1 Introduction

Early marriage, defined as a formal or informal union before the age of 18 years, remains a significant global public health, developmental, and human rights concern. Despite sustained international efforts, an estimated 12 million girls marry each year before reaching adulthood, with the burden concentrated in low‐ and middle‐income countries and humanitarian settings [1, 2]. Contemporary scholarship increasingly recognizes early marriage as a structurally produced practice associated with poverty, gender inequality, limited educational opportunity, weak legal protection, and social insecurity rather than as a solely cultural tradition [3, 4].

The practice is often intensified in fragile and conflict‐affected contexts, where families face economic hardship, displacement, insecurity, and disrupted access to education. Research among refugee and conflict‐affected populations in the Middle East has shown that marriage may be perceived as a strategy to reduce household burden, preserve social reputation, or provide protection for girls in uncertain environments [3, 4]. However, such decisions frequently occur within unequal power relations in which adolescent girls have limited influence over life choices.

Within Palestine, early marriage must be understood within a complex context shaped by prolonged political instability, economic precarity, mobility restrictions, and persistent gendered social expectations. National reports indicate that marriage before age 18 remains present in the West Bank, particularly in rural and socioeconomically marginalized communities [5]. Recent Palestinian and conflict‐related studies further suggest that women's marital trajectories are shaped by interactions between family authority, community norms, and structural constraints affecting education, employment, and autonomy [6, 7, 8, 16, 17]. Family authority refers to the influence of parents, extended relatives, and senior household members on decisions related to marriage timing, partner selection, schooling, mobility, and acceptable female behavior. Community norms reinforce these decisions by linking girls’ conduct to family reputation, protection, gender respectability, and obedience. Structural constraints, including poverty, insecurity, restricted movement, limited educational continuity, and weak employment prospects, further narrow the alternatives available to girls and their families. These forces interact dynamically: economic hardship may strengthen family control over marriage decisions, community expectations may normalize early marriage as protection, and limited access to education or employment may reduce girls’ practical ability to resist or delay marriage. Accordingly, early marriage in Palestine reflects both local sociocultural practices and broader structural pressures that shape girls’ autonomy across the life course.

Extensive evidence links early marriage with adverse short‐ and long‐term outcomes. Cross‐sectional studies from the Middle East and comparable settings associate early marriage with interrupted education, early childbearing, psychological distress, social isolation, reduced decision‐making power, and lower socioeconomic participation [2, 9, 10]. Global evidence also indicates intergenerational consequences, including reduced educational opportunities for children and persistent household vulnerability [11]. Nevertheless, much of the existing literature relies on prevalence estimates or quantitative associations and provides limited understanding of how women themselves interpret these experiences over time.

Qualitative studies have improved understanding of family negotiations, coping strategies, and gender expectations [3, 4, 12]. However, three gaps remain. First, relatively little research has examined long‐term retrospective reflections among women who married early and how meanings evolve across the life course. Second, differences within experiences such as variation by age at marriage, later educational access, or marital trajectory are often underdeveloped analytically. Third, many studies remain descriptive and insufficiently connect empirical narratives to theory, limiting deeper explanation of how agency, power, and structural constraint operate in practice.

These gaps are particularly important in Palestine, where women's lives unfold within intersecting systems of family authority, social norms, and political constraint. Recent scholarship on Palestinian women's marriage experiences, divorce, and economic rights highlights the need for contextually grounded research that centers women's voices and captures both continuity and change across time [7, 8, 13, 14, 15].

Therefore, this study aimed to explore how Palestinian women in the West Bank who married before the age of 18 retrospectively interpret their early marriage experiences and their long‐term effects on health, identity, education, and family life. By focusing on women's lived narratives, the study seeks to contribute analytically grounded evidence on early marriage within conflict‐affected settings.

## 2 Theoretical Framework

This study was guided by an integrated theoretical framework combining Bronfenbrenner's ecological systems theory and feminist theory. These frameworks were applied as analytical lenses rather than descriptive backdrops to explain how early marriage is shaped by interacting social environments, gendered power relations, and changing life‐course experiences. The combined use of these perspectives supports a deeper understanding of early marriage as both structurally embedded and personally experienced [16, 19].

### 2.1 Ecological Systems Perspective

Bronfenbrenner's ecological systems theory conceptualizes development as occurring within nested and interacting systems that range from immediate relationships to wider sociopolitical structures [17, 19]. Within this study, the framework helps explain how participants’ experiences of early marriage were shaped across multiple levels.

At the microsystem level, family relationships were central to marriage decision‐making. Many participants described parents or extended family members arranging marriage with limited adolescent involvement. Statements such as “my family arranged everything” illustrate how authority operated through everyday family interactions, where obedience and compliance were socially normalized. Similar patterns of family‐led decision‐making have been documented in Middle Eastern and humanitarian contexts [1, 9, 10].

At the mesosystem level, the interaction between family expectations and institutions was particularly visible in educational disruption. Several women described leaving school after engagement, marriage, or pregnancy. This suggests that early marriage is shaped not only by household decisions but also by the intersection of family norms with limited institutional support for girls’ continued education. Previous studies have similarly linked child marriage with school discontinuation and restricted life opportunities [8, 11].

At the macrosystem level, broader gender norms, economic precarity, and insecurity may normalize early marriage as an acceptable response to uncertainty. In Palestine, these pressures can be intensified by prolonged political instability, mobility restrictions, and unequal access to resources [4, 11, 18]. Such structural forces shape household decisions while limiting perceived alternatives for girls and families.

Family authority operates at the microsystem level through parental decision‐making and obedience expectations. Community norms operate at the meso‐ and macrosystem levels by defining acceptable female behavior and linking marriage with protection and family reputation. Structural constraints, including poverty, restricted mobility, and limited educational or employment opportunities, narrow the choices available to girls and families [16, 17].

The chronosystem is especially relevant because this study explored retrospective reflections years after marriage. Participants’ narratives showed that meanings changed over time. Experiences initially accepted as normal were later reinterpreted as loss, constraint, or injustice. This temporal dimension indicates that early marriage is not a single event but a life‐course process whose significance evolves through later experiences and opportunities [13].

### 2.2 Feminist Perspective

Although ecological theory explains multilevel influences, feminist theory provides a critical lens for understanding how those systems are gendered. Feminist scholarship emphasizes that power is often reproduced through everyday norms, institutions, and expectations rather than through overt force alone [6, 24].

In this study, participants’ limited involvement in marital decisions reflected unequal authority relations in which decision‐making power often shifted from parental control to marital control. Early marriage may therefore function as a socially sanctioned practice through which girls’ autonomy is constrained, whereas expectations of obedience, caregiving, and early motherhood are reinforced. Comparable findings have been reported in Palestine and neighboring contexts, where girls frequently have reduced influence over marriage timing and partner selection [17, 19].

Feminist analysis also highlights that agency should not be understood as simply absent or present. Several participants described later efforts to resume education, guide their daughters differently, or critically reinterpret their own past experiences. These accounts suggest that agency can emerge within constraint and may expand over time through changing circumstances, knowledge, and social position [20, 21].

### 2.3 Integrated Application to the Present Study

The combination of ecological and feminist perspectives enabled interpretation of early marriage as both structurally constrained and dynamically negotiated. For example, participants married at very young ages often described little understanding of marriage and almost no decision‐making role, illustrating the intersection of developmental vulnerability, family authority, and gendered power. In contrast, those married at older ages sometimes described limited negotiation, although options remained restricted.

Similarly, women who later resumed education often expressed more complex reflections that combined regret with adaptation, whereas those denied later opportunities described more enduring loss. These contrasts demonstrate that experiences of early marriage are not uniform; they are shaped by age at marriage, family context, educational access, and later life opportunities.

Using this integrated framework allowed the study to move beyond descriptive reporting toward explanation, clarifying how power, agency, and structural constraint were enacted in everyday life and reinterpreted across time. This approach strengthens the analytical contribution of the study and aligns theory more closely with participants’ lived experiences.

### 2.4 Research Question

How do Palestinian women who married before age 18 retrospectively interpret their early marriage experiences and their long‐term impact on health, identity, and family life?

## 3 Methods

### 3.1 Research Design

This study employed a qualitative descriptive design within an interpretive framework to explore the lived experiences and long‐term reflections of Palestinian women who were married before the age of 18. A qualitative approach was selected to enable in‐depth exploration of meaning‐making processes within complex sociocultural and political contexts, particularly in relation to sensitive and life‐course experiences. The study was conducted and reported in accordance with the consolidated criteria for reporting qualitative research (COREQ) to ensure methodological transparency and rigor.

### 3.2 Setting and Context

The study was conducted in the Hebron Governorate in the southern West Bank, a region characterized by pronounced socioeconomic disparities, rural–urban variation, and ongoing political instability. These contextual conditions are important, as prior evidence indicates that early marriage in Palestine is shaped by intersecting structural constraints, including poverty, mobility restrictions, and entrenched gender norms [5, 6, 7]. Situating the study within this context allows for a more grounded interpretation of participants’ experiences.

### 3.3 Sampling Strategy and Recruitment

A purposive sampling strategy was employed to recruit participants who could provide rich, information‐dense accounts relevant to the research question. Inclusion criteria required participants to be women who had married before the age of 18, were currently residing in the Hebron Governorate, and were willing to reflect on their experiences retrospectively.

Recruitment was conducted through collaboration with local women's support centers, community‐based organizations, and informal social networks. To address potential selection bias raised by reviewers, recruitment was intentionally diversified across multiple access points, including referrals from social workers and community mediators, rather than relying on a single institutional source. This approach facilitated inclusion of participants with varied educational backgrounds, marital trajectories, and residential contexts (rural and urban).

Ten participants were ultimately recruited. Although this sample size is consistent with qualitative inquiry focused on depth rather than breadth, adequacy was determined based on analytic sufficiency rather than numerical targets.

### 3.4 Sample Adequacy and Saturation

Sample adequacy was assessed using principles of thematic saturation and information power, focusing on the depth, richness, and variability of the data rather than sample size alone [6]. Saturation was evaluated through continuous comparison of emerging codes and themes across interviews. Initial saturation was observed after eight interviews, as no substantially new codes or thematic dimensions were identified. However, two additional interviews were conducted to confirm the stability of the thematic structure and to ensure representation of variation across participants, particularly in relation to age at marriage, education, and residence.

In addition to code repetition, analytic sufficiency was determined through

- Density of coding across transcripts.
- Recurrence of key patterns across cases.
- Identification of both convergent and divergent experiences.

This approach addresses reviewer concerns by demonstrating that saturation was assessed analytically rather than assumed procedurally.

Participant characteristics are detailed in Table 1, which provides contextual information essential for interpreting variability across cases.

**TABLE 1:** Participant demographic characteristics.

Participant code	Current age	Age at marriage	Education level at time of marriage	Current education level	Residence type
P1	25	9	9th grade	Diploma	Rural
P2	23	7	8th grade	No further education	Urban
P3	34	17	10th grade	University	Rural
P4	29	16	9th grade	High school	Urban
P5	30	13	Secondary school	University	Rural
P6	27	10	7th grade	No further education	Rural
P7	33	16	9th grade	Vocational training completed	Urban
P8	30	15	8th grade	Diploma	Urban
P9	27	11	10th grade	Currently in university	Rural
P10	35	16	9th grade	No further education	Rural

### 3.5 Data Collection

Data were collected through semi‐structured, in‐depth interviews conducted in Arabic between [insert timeframe]. Interviews lasted approximately 45–60 min and were conducted either face‐to‐face or via secure online platforms, depending on participant preference and safety considerations.

The interview guide was developed on the basis of a synthesis of empirical literature (Table 2) and the study's theoretical framework. Questions were designed to elicit experiences across multiple domains, including marriage decision‐making, educational trajectories, health and psychosocial impacts, identity formation, and retrospective reflections over time. The guide was reviewed by two qualitative research experts and pilot‐tested with two women who met the inclusion criteria but were not included in the final sample. Minor revisions were made to enhance clarity, cultural sensitivity, and depth of inquiry.

**TABLE 2:** Literature review summary and identified research gaps.

Study	Country	Design	Key findings	Relevance to current study
El Arab and Sagbakken [1]	MENA (multi‐country)	Systematic review	Early marriage driven by gender norms, poverty, and conflict; associated with poor reproductive and mental health outcomes	Establishes regional structural drivers; supports ecological/macrosystem analysis
Girls Not Brides [3]	Global/MENA	Policy + empirical synthesis	Humanitarian crises, insecurity, and displacement increase child marriage risk	Supports conflict‐context analysis and structural pressures
Malik et al. [4]	Palestine	Mixed‐method narrative study	Palestinian women's marital trajectories shaped by social and gendered inequalities	Supports Palestinian life‐course and gender context
Hattab and Abualrob [5]	Palestine	Policy/Legal analysis	Women's marriage and economic rights constrained by structural inequalities	Supports autonomy, rights, and macrosystem analysis
Jones et al. [6]	Global	Methodological/Theoretical paper	Feminist approaches strengthen analysis of child marriage and gendered power	Supports feminist framework and interpretation
Montazeri et al. [8]	Iran	Cross‐sectional qualitative‐informed study	Early marriage linked to reduced autonomy, mental distress, and limited educational attainment	Supports psychosocial and autonomy‐related findings
DeJong et al. [9]	Lebanon (Syrian refugees)	Qualitative	Families use early marriage as protection strategy in conflict; girls excluded from decision‐making	Supports microsystem (family control) and conflict relevance
Mourtada et al. [10]	Jordan (Syrian refugees)	Mixed‐methods	Economic insecurity and social norms increase child marriage; education interruption common	Supports mesosystem (education disruption) and structural pressures
Veronese et al. [12]	Palestine	Qualitative	Women demonstrate resilience despite conflict‐related structural constraints	Supports resilience discussion and adaptive coping
Meler and Marnin‐Distelfeld [13]	Palestine (Arab population)	Qualitative	Early marriage shapes identity, motherhood roles, and educational discontinuity	Supports life‐course and identity‐related findings
Alzoubi et al. [14]	Jordan	Cross‐sectional	Early marriage associated with poor mental health and social isolation	Supports emotional burden and isolation findings
Ahmed et al. [15]	Global	Systematic review	Adolescents may lack full cognitive/emotional maturity for marital roles	Supports developmental vulnerability argument
Figueiredo and Silvestre [16]	Portugal	Cross‐sectional study	Institutionalization was associated with difficulties in emotion regulation, attention, and sleep hygiene among children	Supports the argument that disrupted childhood environments may affect psychosocial development, emotional regulation, and later vulnerability
Miller‐Graff and Cummings, [17]	Gaza/Palestine or relevant setting	Randomized controlled trial/verify exact design	Family‐based intervention evidence shows the importance of family systems and psychosocial support in conflict‐affected settings	Supports the need to analyze family systems, adversity, and psychosocial support in Palestinian or conflict‐affected contexts

All interviews were audio‐recorded with participants’ informed consent and transcribed verbatim. Given the importance of linguistic nuance in qualitative research, transcripts were translated into English using a rigorous process involving bilingual verification and back‐checking. The research team critically examined translation choices to ensure that meanings, cultural expressions, and emotional tone were preserved, addressing reviewer concerns regarding the absence of reflexive consideration of translation.

### 3.6 Data Analysis

Data were analyzed using reflexive thematic analysis, following the approach developed by Braun and Clarke [19], with an explicitly interpretive and iterative orientation. Analysis did not follow a strictly linear sequence but involved continuous movement between data, codes, themes, and theoretical interpretation.

The analytic process began with immersion in the data through repeated reading of transcripts. Initial coding was conducted inductively, capturing both semantic content (explicit statements) and latent meanings (underlying assumptions and power relations). Codes were then compared across transcripts to identify patterns, similarities, and differences.

Theme development involved clustering related codes into broader conceptual categories. Importantly, themes were not treated as purely descriptive summaries but were interpreted through the study's theoretical framework. This included mapping themes onto ecological system levels and examining how gendered power relations shaped participants’ experiences.

To enhance analytical rigor, particular attention was given to

- **Variation across cases**, including differences related to age at marriage, education, and residence.
- **Negative cases and contradictions**, where participants’ experiences diverged from dominant patterns.
- **Depth of interpretation**, moving beyond paraphrasing toward explanation of underlying mechanisms.

For example, although many participants described limited agency in marriage decisions, some reported later‐life efforts to regain autonomy, such as returning to education or influencing their daughters’ life choices. These cases were analyzed as forms of evolving agency rather than exceptions to the rule.

Theoretical integration occurred during later stages of analysis, where themes were interpreted in relation to ecological systems and feminist concepts of power, rather than imposing theory at the outset. This approach ensured that theory functioned as an analytical lens rather than a descriptive framework.

### 3.7 Rigor and Trustworthiness

Several strategies were employed to enhance methodological rigor.

Credibility was supported through member checking, whereby summary interpretations were shared with five participants, all of whom confirmed that the findings accurately reflected their experiences. Peer debriefing was conducted with two independent qualitative researchers who reviewed coding decisions and thematic interpretations, providing critical feedback on coherence and potential bias.

Dependability and confirmability were ensured through the maintenance of an audit trail documenting all stages of data collection and analysis, including coding revisions and analytic decisions. Reflexive journaling was used throughout the research process to critically examine the researchers’ assumptions, positionality, and potential influence on interpretation. Transferability was enhanced through detailed contextual descriptions of participants and setting, as presented in Table 2, allowing readers to assess applicability to other contexts.

### 3.8 Reflexivity

Given the researchers’ cultural proximity to participants, reflexivity was an essential component of the study. Although shared cultural background facilitated rapport and depth of disclosure, it also required careful consideration of potential biases in interpretation. The research team engaged in ongoing reflexive dialogue and documentation to ensure that findings were grounded in participants’ narratives rather than researcher assumptions.

### 3.9 Ethical Considerations

Ethical approval was obtained from the Palestine Polytechnic University Research Ethics Committee (Approval No. REC‐22‐2025). All participants provided written informed consent after receiving detailed information about the study's purpose, procedures, and their rights.

Given the sensitive nature of the topic, several measures were implemented to protect participants. Interviews were conducted in private and safe environments by a trained female researcher. Participants were informed of their right to decline answering any question or to withdraw from the study at any time without consequence. Following the interviews, participants were offered emotional support and, when necessary, referrals to local psychosocial services.

Confidentiality was strictly maintained through the use of pseudonyms, secure data storage, and restricted access to identifiable information.

## 4 Results

This analysis examined how participants’ narratives reflected the reproduction, negotiation, and occasional resistance of gendered and structural constraints. Across cases, early marriage emerged not as a single event but as a process shaped by family authority, developmental vulnerability, and later reinterpretation. Three interrelated themes were identified: (1) negotiated and constrained agency in marital decision‐making; (2) cumulative postmarital consequences; and (3) evolving reinterpretations of self and life trajectory.

### 4.1 Negotiated and Constrained Agency in Marital Decision‐Making

Participants’ accounts did not indicate a simple absence of agency. Rather, agency operated along a continuum shaped by age, family power, and perceived alternatives. Some women described complete exclusion from decisions, whereas others described conditional agreement shaped by economic pressure or emotional obligation.

For several participants, statements such as “My parents arranged everything” reflected more than lack of consultation. They illustrated family systems in which obedience was expected and parental authority was socially normalized. In these cases, power was exercised through routine family relations rather than explicit force.

A contrasting example was P5, who described accepting marriage to avoid burdening her family financially. This account suggests constrained agency: Agreement was expressed, but within narrow economic and relational limits. Consent therefore appeared less as free choice and more as adaptive compliance.

Variation by age at marriage was particularly clear. Participants married at ages **7–10 years** reported little understanding of marriage and almost no involvement in decisions. By contrast, those married at **15–17 years** described greater awareness and occasional attempts to negotiate, although meaningful options remained limited. This contrast indicates that agency was shaped by both developmental stage and gendered family authority.

Overall, marital decision‐making was relational and hierarchical, influenced by family expectations, economic insecurity, and the participant's age at marriage.

### 4.2 Interconnected and Cumulative Postmarital Consequences

Participants rarely described consequences as isolated physical, emotional, or social problems. Instead, these experiences were interconnected and often intensified over time.

#### 4.2.1 Physical Strain Within Gendered Roles

Accounts of fatigue, repeated pregnancy, childbirth complications, and chronic pain were common. These experiences were closely linked to early transition into motherhood and domestic labor. Women married at younger ages often described more severe strain, suggesting that biological vulnerability intersected with expectations of immediate reproductive and household responsibilities.

Several narratives also indicated limited autonomy in health‐related decisions, including delayed care‐seeking or dependence on others for access to services. Physical burden was therefore embedded within broader structural constraints rather than solely individual health status.

#### 4.2.2 Social Isolation and Loss of Support Networks

Social isolation emerged as both an outcome and a mechanism of dependency. Marriage frequently interrupted schooling, reduced contact with peers, and, in some cases, required relocation to the husband's household. These changes narrowed participants’ access to support, identity, and independence.

Variation was again notable. Participants who later resumed education or maintained learning pathways (e.g., P3, P5, and P9) described wider social networks and greater confidence. Those denied such opportunities more often described loneliness and restricted daily life. This contrast suggests that educational continuity functioned as a protective factor.

#### 4.2.3 Emotional Burden and Everyday Negotiation

Participants described anxiety, pressure, sadness, and self‐doubt. However, emotional burden was often tied to ongoing efforts to meet expectations within marital households. For example, one participant stated, “I always try to prove myself,” indicating active attempts to secure acceptance through performance of expected roles.

Another participant said, “I feel like I'm going to explode,” illustrating the emotional cost of sustained pressure. These examples show how distress was linked to unequal domestic expectations and limited room for self‐expression.

Some women also described later efforts to renegotiate roles, seek education, or increase independence. These cases suggest that agency could re‐emerge within constrained settings, although unevenly and often gradually.

### 4.3 Temporal Reinterpretation and Disrupted Life Trajectories

A major finding was that meanings attached to early marriage changed over time. Experiences that were initially normalized were later reconsidered through adulthood, motherhood, or later educational exposure.

#### 4.3.1 Regret as Later Recognition of Lost Opportunity

Many participants expressed regret, especially regarding interrupted education. Statements such as “I would finish my education” reflected more than personal disappointment; they indicated later recognition of opportunities that had been structurally restricted.

This reinterpretation varied by later life trajectory. Women who reentered education or completed vocational training often expressed mixed narratives of regret and adaptation. In contrast, those who remained excluded from education described more definitive loss.

#### 4.3.2 Education as Agency and Resistance

Education was repeatedly described not only as a missed opportunity but as a route to autonomy and dignity. One participant stated, “Education is a woman's only weapon.” This framing positioned education as both practical resource and symbolic resistance.

Several women also described wanting different futures for their daughters and explicitly rejecting early marriage for the next generation. These accounts suggest that retrospective reflection could translate into intergenerational change.

#### 4.3.3 Variation and Contradictions

Although regret and constraint were common, experiences were not uniform. A small number of participants identified positive aspects of their current lives or expressed partial acceptance of past events. These accounts did not negate dominant themes but demonstrated that outcomes were shaped by later opportunities, family relationships, and personal circumstances.

Taken together, the findings indicate that early marriage among Palestinian women was a dynamic life‐course process. Agency was constrained but not absent, harms were cumulative rather than isolated, and meanings evolved through reflection, adaptation, and, in some cases, resistance.

## 5 Discussion

These findings show that family authority, community norms, and structural constraints worked together. Family authority shaped marriage decisions, community norms justified early marriage as protection and reputation preservation, and poverty, restricted mobility, and limited schooling reduced alternatives. Thus, early marriage appeared not as a free individual choice but as a socially normalized response to insecurity that affected girls’ education, health, and autonomy [16, 17].

This study provides a refined understanding of early marriage among Palestinian women, demonstrating that it functions not simply as a cultural tradition but as a multilevel social process shaped by family authority, gender norms, economic precarity, and constrained future opportunities. By foregrounding retrospective narratives, the study extends literature that has often emphasized prevalence, legal status, or short‐term outcomes [1, 2, 3, 4, 5]. Participants’ accounts showed that early marriage was associated with constrained agency, interrupted education, cumulative psychosocial burden, and changing interpretations across the life course. At the same time, experiences were not uniform; variation, adaptation, and selective resistance were also evident.

A central contribution of this study is the finding that agency was neither wholly absent nor fully autonomous. Rather than a binary distinction between coercion and choice, participants described decisions shaped by family pressure, financial strain, emotional obligation, and limited alternatives. Statements such as “my parents arranged everything” reflected authority embedded in everyday family relations where obedience was expected. In these accounts, power operated through normalization rather than overt force.

This interpretation is consistent with evidence from Jordan, Lebanon, and other humanitarian settings where marriage decisions are often framed as family protection strategies during insecurity [1, 3, 9, 10]. Similar patterns have been described in Palestine, where gender norms and family authority can reduce girls’ influence over marital timing and partner selection [17, 22]. Feminist scholarship also emphasizes that consent must be interpreted within surrounding social conditions rather than as an isolated individual act [6, 23, 24].

Variation by age at marriage was especially important. Participants married at ages 7–10 years described little understanding of marriage and almost no participation in decisions. By contrast, those married at ages 15–17 years sometimes described partial awareness or limited negotiation, although meaningful alternatives remained constrained. This supports developmental evidence indicating that adolescents’ cognitive and emotional maturity affects decision‐making capacity [15, 16, 25]. Thus, agency in early marriage contexts appears relational, conditional, and strongly mediated by age.

The findings suggest that early marriage should be understood less as a single event and more as a pathway generating cumulative disadvantage. Educational disruption was particularly prominent. Participants who left school described later dependence, reduced confidence, narrower social networks, and persistent regret. In contrast, women who resumed education or vocational training often described stronger self‐worth and broader life options.

This pattern aligns with global evidence showing that child marriage is associated with lower educational attainment, reduced labor participation, and persistent household vulnerability [2, 8, 18]. Research among migrant and displaced women similarly shows that interrupted education can shape long‐term social and economic trajectories [25, 26]. In the present study, however, education also held symbolic meaning. Several women described learning as dignity, voice, and protection, suggesting that schooling represented both practical opportunity and social recognition.

Physical strain was also common. Narratives of repeated pregnancy, childbirth complications, chronic fatigue, and domestic labor were especially marked among women married at younger ages. This supports evidence linking early marriage to adolescent reproductive risk and poorer maternal outcomes [7, 14, 23, 27]. Importantly, participants also described needing permission or support to seek care, indicating that health burdens were intensified by limited autonomy rather than by age alone.

Participants’ emotional experiences were closely connected to unequal domestic expectations. Statements such as “I always try to prove myself” suggested continuous efforts to gain legitimacy as wives, daughters‐in‐law, or mothers. Emotional distress therefore appeared less as an individual pathology and more as a response to unequal responsibility combined with limited authority.

This interpretation is consistent with research on Palestinian women's resilience under structural pressure, where emotional strain often coexists with adaptation and persistence [12]. Similar findings have been reported in Jordan and Oman, where early marriage was associated with anxiety, isolation, and reduced coping capacity [11, 14, 28]. By situating distress within household power relations, the present findings move beyond individualized explanations toward a more relational account.

A distinctive contribution of this study lies in showing how meanings attached to early marriage changed over time. Many participants initially described marriage as normal or unavoidable but later reconsidered it through adulthood, motherhood, or exposure to education. Regret regarding interrupted schooling was particularly strong. Statements such as “I would finish my education” reflected later recognition of opportunities that had been structurally foreclosed.

These findings align with life‐course research showing that individuals reinterpret earlier experiences as their social positions change [13, 20, 29]. However, reinterpretation was shaped by later opportunity structures. Women who entered university, vocational training, or employment often combined regret with adaptation. Those who remained excluded described more definitive loss. This indicates that retrospective meaning‐making is socially patterned rather than purely psychological.

Education also emerged as a site of resistance. Several participants described wanting different futures for their daughters and explicitly rejecting early marriage for the next generation. This intergenerational stance suggests that constrained agency in one generation may still contribute to normative change in the next [2, 18, 30].

Although regret and hardship were common, the findings did not support a uniform narrative of victimhood. Some participants described stable relationships, pride in motherhood, or satisfaction with aspects of their current lives. These contradictory accounts are analytically important because they show that structural constraint does not determine identical outcomes.

Variation was linked to age at marriage, educational pathways, family relationships, and later access to opportunity. Similar heterogeneity has been documented in Ethiopia, refugee settings, and migrant populations, where outcomes vary according to household support, schooling continuity, and socioeconomic resources [21, 22, 25, 28]. Recognizing this diversity strengthens interpretation by showing that structural conditions shape probabilities, not destinies.

The integrated theoretical framework helps explain how early marriage was reproduced across interacting systems. At the microsystem level, family authority shaped immediate decisions. At the mesosystem level, marriage disrupted schooling and peer relationships. At the macrosystem level, gender norms, economic inequality, weak enforcement mechanisms, and political instability narrowed perceived alternatives [4, 5, 8, 11]. In Palestine, these pressures are further intensified by broader structural restrictions affecting mobility, employment, and security [27, 30].

The chronosystem dimension was also evident. Participants’ narratives showed that meanings evolved across time, especially after motherhood, educational reentry, or later comparison with peers [19]. Feminist analysis further clarifies that power frequently operated through obligation, duty, and normalized expectations rather than direct coercion [6, 24]. Yet women's later pursuit of education, reinterpretation of past events, and protective aspirations for daughters indicate that agency can be reconstructed within constraint.

### 5.1 Implications for Policy and Practice

The findings suggest that responses to early marriage should move beyond legal prohibition alone. Although minimum‐age laws are important, participants’ narratives indicate that decisions were also shaped by poverty, insecurity, and family reputation. Effective prevention therefore requires integrated strategies combining legal enforcement with economic support, community engagement, and girls’ educational retention.

Educational continuity should be prioritized through reentry pathways for married or previously married girls, flexible schooling, and vocational opportunities. Participants repeatedly framed education as transformative, reinforcing evidence that schooling is central to empowerment and delayed marriage.

Healthcare systems should also address the needs of early married adolescents and young women through accessible reproductive health, mental health, and confidential counseling services. In conflict‐affected settings, these services must remain functional despite mobility barriers and resource constraints.

### 5.2 Strengths and Limitations

This study contributes rare qualitative evidence from Palestine, where research has often emphasized prevalence rather than lived experience. The retrospective design enabled exploration of how meanings shift across time, while attention to variation strengthened analytical depth.

Several limitations should be acknowledged. The sample was small and drawn from one governorate, which may limit transferability. Retrospective narratives may also be shaped by later experiences and current circumstances. In addition, participants willing to speak may differ from those who declined. Nevertheless, the consistency of core themes alongside meaningful contradictions supports the credibility of the findings.

## 6 Conclusion

Early marriage among Palestinian women emerged as a structurally embedded and gendered life‐course process rather than a single marital event. Participants’ narratives showed constrained agency, disrupted education, cumulative psychosocial burden, and evolving interpretations over time. Yet they also revealed resilience, adaptive meaning‐making, and aspirations for different futures for the next generation. These findings underscore the need for contextually grounded interventions addressing family power relations, educational exclusion, poverty, and broader structural determinants.

## Author Contributions

**Nesreen Alqaissi**: conceptualization, methodology, investigation, data collection, literature review, writing – original draft preparation, and writing – review and editing. **Mohammad Qtait**: conceptualization, methodology, supervision, formal analysis, validation, project administration, writing – review and editing, and correspondence with the journal. Both authors read and approved the final version of the manuscript and agreed to be accountable for all aspects of the work.

## Funding

The authors have nothing to report.

## Conflicts of Interest

The authors declare no conflicts of interest.

## Data Availability

The data that support the findings of this study are available from the corresponding author upon reasonable request. The data are not publicly available due to ethical restrictions, including the protection of participant confidentiality and institutional regulations governing human subject research.
